# Probing binding hot spots at protein–RNA recognition sites

**DOI:** 10.1093/nar/gkv876

**Published:** 2015-09-13

**Authors:** Amita Barik, Chandran Nithin, Naga Bhushana Rao Karampudi, Sunandan Mukherjee, Ranjit Prasad Bahadur

**Affiliations:** 1 Computational Structural Biology Laboratory, Department of Biotechnology, Indian Institute of Technology Kharagpur, Kharagpur-721302, India; 2 Advanced Technology Development Centre, Indian Institute of Technology Kharagpur, Kharagpur-721302, India

## Abstract

We use evolutionary conservation derived from structure alignment of polypeptide sequences along with structural and physicochemical attributes of protein–RNA interfaces to probe the binding hot spots at protein–RNA recognition sites. We find that the degree of conservation varies across the RNA binding proteins; some evolve rapidly compared to others. Additionally, irrespective of the structural class of the complexes, residues at the RNA binding sites are evolutionary better conserved than those at the solvent exposed surfaces. For recognitions involving duplex RNA, residues interacting with the major groove are better conserved than those interacting with the minor groove. We identify multi-interface residues participating simultaneously in protein–protein and protein–RNA interfaces in complexes where more than one polypeptide is involved in RNA recognition, and show that they are better conserved compared to any other RNA binding residues. We find that the residues at water preservation site are better conserved than those at hydrated or at dehydrated sites. Finally, we develop a Random Forests model using structural and physicochemical attributes for predicting binding hot spots. The model accurately predicts 80% of the instances of experimental ΔΔG values in a particular class, and provides a stepping-stone towards the engineering of protein–RNA recognition sites with desired affinity.

## INTRODUCTION

Majority of cellular functions are governed by macromolecular interactions, and thus macromolecules are under constant evolutionary pressure for selecting their partners in a crowded cellular environment. Consequently, interacting surfaces experience relatively more evolutionary pressure and undergo fewer mutations than the surfaces that are in contact with the bulk solvent, a phenomenon which is often observed in protein–protein interfaces (1–4). Mutagenesis studies revealed that the substitution of the functional groups at the recognition sites may dramatically change the binding affinity (5,6). Structurally conserved residues have often been used to determine the oligomeric state of the proteins (7), and are subject to the drug targets (5). They have also been extensively used to discriminate between the transient and the obligate protein–protein complexes (8), as well as between the biological contact and the crystal-packing protein–protein interfaces (9,10). Bahadur and Janin (11) also elucidated the role of structurally conserved residues in viral capsid assemblies. Furthermore, it has been shown that the evolutionary conserved residues often contribute significantly to the binding free energy (2,10,12); however, they are not distributed evenly at the recognition site (5,11,13).

Proteins specifically recognize their partner RNA in a crowded cellular environment, and the resultant protein–RNA interactions govern many cellular processes (14). Protein–RNA complexes are stabilized by the non-covalent interactions, and interacting polypeptide and nucleotide chains are always under the evolutionary constraints. Recent advancement in the structure determination of protein–RNA complexes revealed the principle of RNA recognition by individual RNA Binding Domains (RBDs). The recognition of RNA by RBDs suggests additional modes of RNA recognition by combination and cooperation of these interactions (15). In spite of these advancements, prediction of the binding energy hot spots at the protein–RNA recognition sites based on the sequence and structural information is still elusive.

To examine the evolutionary constraints experienced by the polypeptide chain that recognizes RNA, we calculated the Shannon entropy in the aligned sequences of 211 polypeptide chains in 145 protein–RNA complexes. We find that some RNA binding proteins (RBPs) evolve faster compared to others. Entropy values are correlated with the three-dimensional location of the residues in the complex structure. Residues buried at the protein interior are found to be the most conserved ones followed by the residues at the protein–RNA interfaces and the residues at the solvent exposed surfaces. Moreover, where the RNA recognition is governed by more than one polypeptide chain, we find the residues present simultaneously at the protein–protein and the protein–RNA interfaces are the most conserved followed by the residues present either at the protein–RNA interfaces or at the protein–protein interfaces. We show the residues interacting with the major groove of the duplex RNA are better conserved than those interacting with the minor groove. Additionally, we observe that the residues at the WP site (described in the results section) are the most conserved followed by the residues at the WH site and the residues at the WD site. Finally, using the sequence and structural attributes of the protein–RNA interfaces, we have developed a model using Random Forests to predict the change in binding free energy (ΔΔG) obtained through alanine scanning mutagenesis experiments. Our model accurately predicts 80% of the instances of ΔΔG values. The method is implemented in a web server ‘HotSPRing (http://www.csb.iitkgp.ernet.in/applications/HotSPRing/main)’ to predict the host spots in RBPs.

## MATERIALS AND METHODS

### Data set of the protein–RNA complexes

Atomic structures of protein–RNA complexes with resolution better than 3 Å were curated from the Protein Data Bank (PDB) (16). Complexes having protein chain of at least 30 amino acids and RNA chain of at least 5 nucleotides were retained in the data set. To make the data set non-redundant, we performed pairwise sequence alignment for all the entries in the data set using BLAST (17). For the protein components in two complexes with more than 35% sequence identity, the one with the better resolution was kept. Additionally, we retained the complexes with homologous protein chains if they bind different RNA sequences, as the removal of one of them would result to the loss of diversity of interactions shown by them. We also performed the pairwise sequence alignment using structural superposition in PDBeFold (18). The sequence identity values obtained using structural superposition were closely similar to those obtained using BLAST. The final data set consists of 152 protein–RNA complexes. Following Bahadur *et al*. (19), the data set is divided into four different classes based on the type of the RNA associated with the proteins: (A) complexes with tRNA, (B) complexes with ribosomal proteins, (C) complexes with duplex RNA and (D) complexes with single-stranded RNA. The data set includes 39 complexes where the recognition of the RNA molecule involves more than one polypeptide chain. In these complexes, the residues in each protomer may appear in protein–protein interfaces (PP), or in protein–RNA interfaces (PR) or in both (PP+PR).

### Interior, interface and surface residues

Protein–RNA interface is defined as a set of amino acid residues and nucleotides that loses Solvent Accessible Surface Area (SASA) in complexation. The interior of the protein comprises of completely buried residues having relative SASA ≤ 5%. The relative SASA is calculated by taking the ratio of the SASA in the complex to the SASA in the extended conformation in Ala-X-Ala form, where X is the residue of concern. Residues that are not part of the interface or the protein interior are assigned as the surface residues.

### Multiple sequence alignment

Multiple sequence alignments of the protein chain of 152 complexes were taken from the HSSP (Homology-Derived Secondary Structure of Proteins) database (20). Usually, the HSSP database provides a list of structural homologs for each PDB entry. However, for some of our entries, this information is missing in the HSSP database. We discarded the sequences that have less than 45% identity with the corresponding entry in the PDB (HSSP parameter %ID < 45), and have more than five missing residues (HSSP parameter LALI < ILAS-IFIR-5) (11). Query sequences having more than six aligned sequences and satisfying the above criteria were retained. Finally we assembled 145 protein–RNA complexes comprising of 203 polypeptide chains satisfying the above criteria. The PDB entries having non-identical protein chains were treated separately.

### Calculation of sequence entropy

Shannon entropy (21) was used to characterize the residue variability at each sequence position of a set of aligned amino acid sequences. If *n_k_* of the N aligned sequences have an amino acid residue of type *k* at position *i*, the frequency of type *k* is *p_k_* = *n_k_/N*, and the sequence entropy is given as:

(1) \begin{equation*} S(i) = - \sum\nolimits _k p_k \;\ln \;p_k \end{equation*}

In the current implementation, the summation is made over 21 residue types, where the type 21 is a gap in the alignment. The entropy S(*i*) varies between 0.0 (at positions that are fully conserved) and ln 21, ∼3.0 (at positions where all 21 types are equally represented in the aligned sequences). In general, S(*i*) depends on the number (N) of aligned sequences and their overall divergence. To correct that dependency, normalized entropies were calculated as:

(2) \begin{equation*} s(i) = {\rm S}(i)/ < {\rm S} >\end{equation*}

where <S> is the mean value of S(*i*) taken over the entire polypeptide chain.

### Probing binding hot spots

Alanine scanning mutagenesis data were curated from the literature. The change in free energy of binding (ΔΔG) was divided into five classes: (i) ≤−1.0; (ii) −1.0 to ≤0.2; (iii) 0.2 to ≤1.0; (iv) 1.0 to ≤2.0 and (v) >2.0 kcal/mol. To avoid the over fitting, the limits for each class were made based on the frequency distribution of the data set. To predict the class of ΔΔG for a given mutation, we have developed a model using Random Forests (RF) (22) implemented in Scikit-learn (23) version 0.15, a module in Python programming language. The mutation data set was split into subsets containing 46 and 19 instances for the training and the test sets, respectively. Test data were chosen randomly by using the subset program of LIBSVM (24). The following parameters were used to train the model: sequence entropy; LD index (Local Density index, calculated following (19)); ΔASA (change in SASA of a residue in the bound structure compared to its unbound form) for the whole residue and the side chain; hydrogen bonds (H-bond, calculated following (25)); Salt-bridges (calculated following (26)); C_α_-rmsd (root mean squared displacement of C_α_ atoms calculated by superposing the unbound and the bound structures); stacking interactions (π-π and π-cation interactions that can occur between the side-chains of Tyr, Trp, Phe, His, Arg and the RNA bases, and are calculated following (27)); classification of mutation based on change in hydrophobicity.

## RESULTS

### Evolution of polypeptide chain in contact with RNA in protein–RNA complexes

The list of 145 protein–RNA complexes used in this study is reported in Supplementary Table S1. In these complexes, the length of the polypeptide chain varies from 44 to 1264 residues. Class A contains 30 complexes including 42 polypeptide chains, class B contains 10 complexes including 12 polypeptide chains, class C contains 43 complexes including 65 polypeptide chains and class D contains 62 complexes including 84 polypeptide chains. <S> values calculated for each of the polypeptide chain are given in the Supplementary Table S1. For biological assemblies containing symmetry related identical polypeptide chains, <S> is calculated only for one chain.

**Figure 1.**
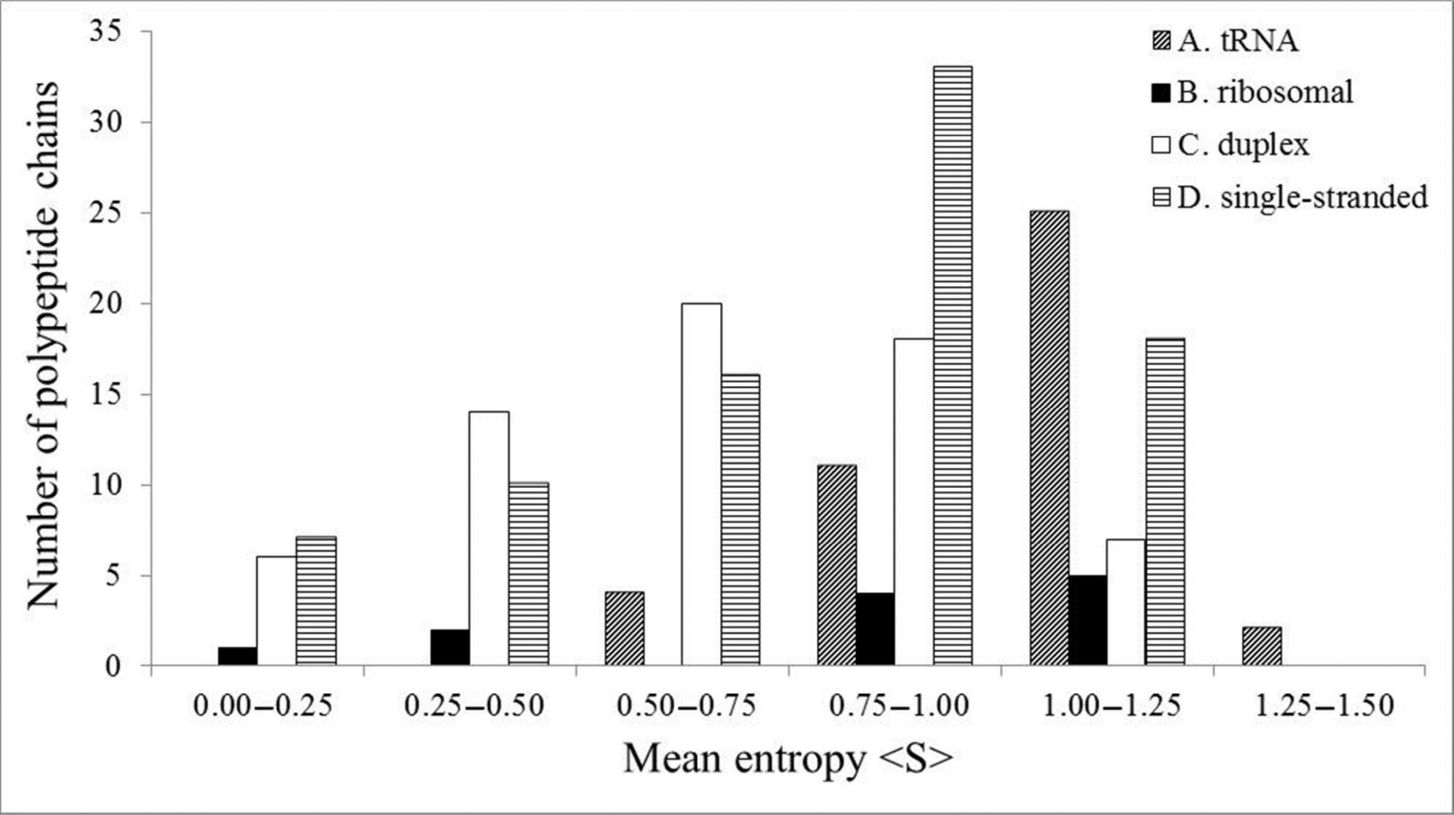
The distribution of mean sequence entropy <S> of different polypeptide chains of protein–RNA complexes.

<S> varies from 0.08 to 1.28 in the entire data set (Figure 1). While the ribosomal protein S6 experience the highest evolutionary pressure (<S> = 0.08), the prolyl-tRNA synthetase experience the lowest evolutionary pressure (<S> = 1.28) (Supplementary Table S1). These suggest that some RBPs evolve rapidly compared to others. In class A, majority of the aminoacyl tRNA synthetases evolve rapidly (<S> ≥ 1.0), while in the rest of the three classes, majority of the proteins evolve slowly (<S> < 1.0).

### Evolution of interior, interface and surface

**Figure 2.**
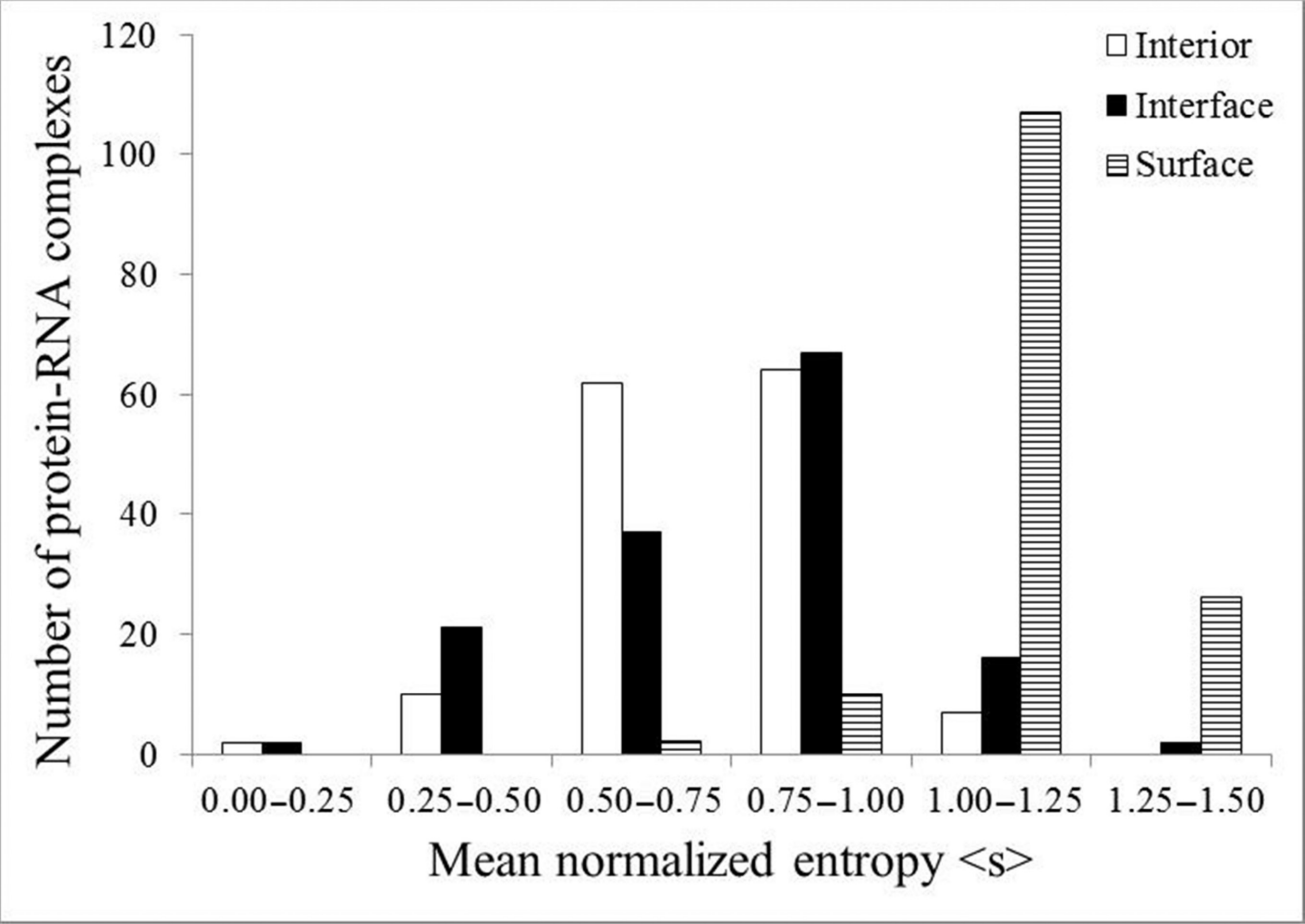
The mean normalized sequence entropy of interior, interface and surface residues in different classes of protein–RNA complexes.

On an average, about 63% residues in a RBP are exposed to the solvent molecules, while only 11% are involved in contact with the RNA (Table 1). The rest are found at the protein interior, providing stability to the folded polypeptide. This trend is essentially similar among the different classes of protein–RNA complexes. A comparison among the mean normalized entropies (<s>) of the residues located at the protein interior, at the interface and at the solvent exposed surface is shown in Table 1. In general, residues belonging to the protein interior have the lowest <s> (0.75), followed by the residues at the protein–RNA interface (<s> = 0.78) and the residues at the solvent exposed surface (<s> = 1.13). This implies that the interface residues are better conserved than the solvent exposed surface residues, while the residues at the protein interior are the most conserved. This trend is essentially similar in all classes, except in class D. Here, the polypeptide chains bind single-stranded RNAs, and the residues at the interface are better conserved (<s> = 0.70) than the residues both at the protein interior (<s> = 0.73) and at the solvent exposed surface (<s> = 1.14). Although the <s> in three different regions differs significantly, their distribution overlaps (Figure 2). Moreover, the <s> of interior and interface are within one σ (standard deviation) range. To examine any significant difference of <s> among interior, interface and surface, t-test was performed for paired samples. While the *P*-value (9.8E-28) shows a significant difference between the evolution of the interface and the surface residues, the evolution of the interior and the interface residues are almost similar (*P* = 0.07).

**Table 1.** Sequence entropy of interior, interface and surface residues

Parameters	Protein–RNA				All	Protein–DNA^a^
	tRNA	ribosomal	duplex	single-stranded		
No. of complexes	30	10	43	62	145	110
No. of aligned sequences	42	12	65	84	203	
% residues in						
interior	29	19	26	24	26	22
interface	10	21	11	11	11	17
surface	61	60	63	65	63	61
Mean <s>						
interior	0.75 ± 0.11	0.89 ± 0.09	0.75 ± 0.23	0.73 ± 0.18	0.75 ± 0.18	0.68 ± 0.17
interface	0.85 ± 0.18	0.90 ± 0.15	0.80 ± 0.25	0.70 ± 0.24	0.78 ± 0.24	0.66 ± 0.22
surface	1.15 ± 0.08	1.04 ± 0.18	1.13 ± 0.11	1.15 ± 0.12	1.13 ± 0.12	1.21 ± 0.11

^a^Calculated on a data set of 110 protein–DNA complexes taken from (30) satisfying the condition mentioned in Materials and Methods section.

**Figure 3.**
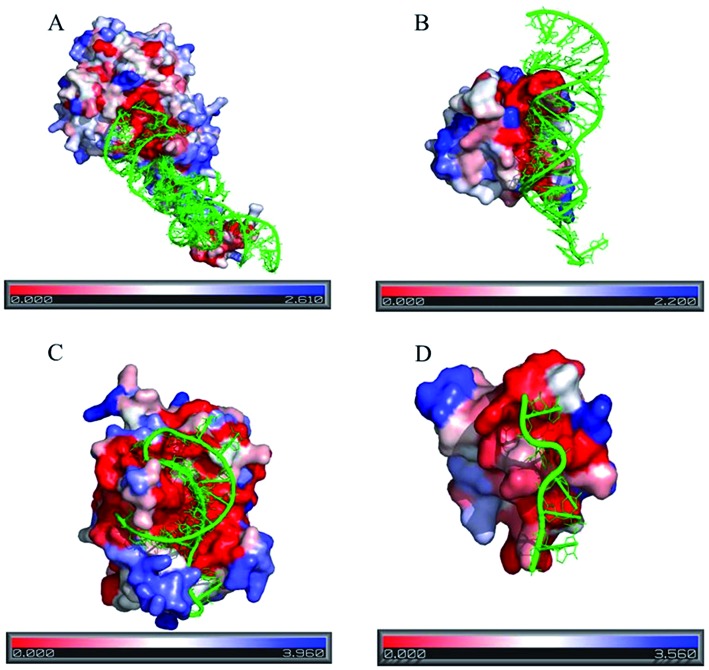
Conservation of the amino acid residues in protein–RNA complexes in four different classes. Residue conservation is mapped at the protein surface with the colour code provided at the bottom. Red stands for the maximum conservation (lowest <s>), and blue stands for the minimum conservation (highest <s>). The RNA backbone is shown in cartoon and coloured green. (**A**) The crystal structure of the TiaS-tRNA (Ile2)-ATP complex (PDB id: 3AMT), (**B**) The crystal structure of the ribosomal protein L25 complexed with 5S rRNA fragment (PDB id: 1DFU), (**C**) The crystal structure of CRISPR endoribonuclease Cse3 bound to 20 nt RNA (PDB id: 2Y8W) and (**D**) The crystal structure of MBNL1 tandem zinc finger 3 and 4 domain complexed with CGCUGU RNA (PDB id: 3D2S).

Figure 3 illustrates the distribution of the residue conservation at the protein surface in four different classes of protein–RNA complexes. In all the four structures, the protein surface that binds the RNA is uniformly coloured red, indicating highly conserved region. Moreover, in all these structures, protein surfaces have noticeable concavity, which is an essential feature for RNA recognition (19).

### Relative conservation of amino acid residues

**Figure 4.**
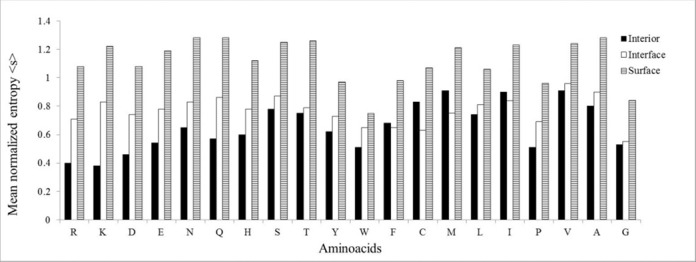
The mean normalized entropy <s> of different amino acid residues in interior, interface and surface.

The relative conservation of each amino acid residue at protein interior, interface and solvent exposed surface is shown in Figure 4. The degree of conservation of most of the residue is almost similar at the protein–RNA interface and at the protein interior. The exceptions are the charged residues, which are better conserved at the interior where they are very rare, than at the interface where they are abundant. At the interface, noticeably the aromatic amino acid residues along with Gly, Cys and Arg are better conserved than any other residues. Aromatic residues along with Arg contribute significantly to the stacking interactions with the RNA bases (28). At the protein surface, neutral polar amino acid residues (Asn, Gln, Thr, Ser) along with Ala are frequently mutated compared to others. <s> values of 20 amino acid residues at protein interior, interface and surface are given in the Supplementary Table S2. The propensity values show that all the amino acid residues at the protein surface evolve faster than at the interface or at the interior. Moreover, except four residues (Cys, Ile, Met and Phe), all others are better conserved at the interior than at the interface.

**Figure 5.**
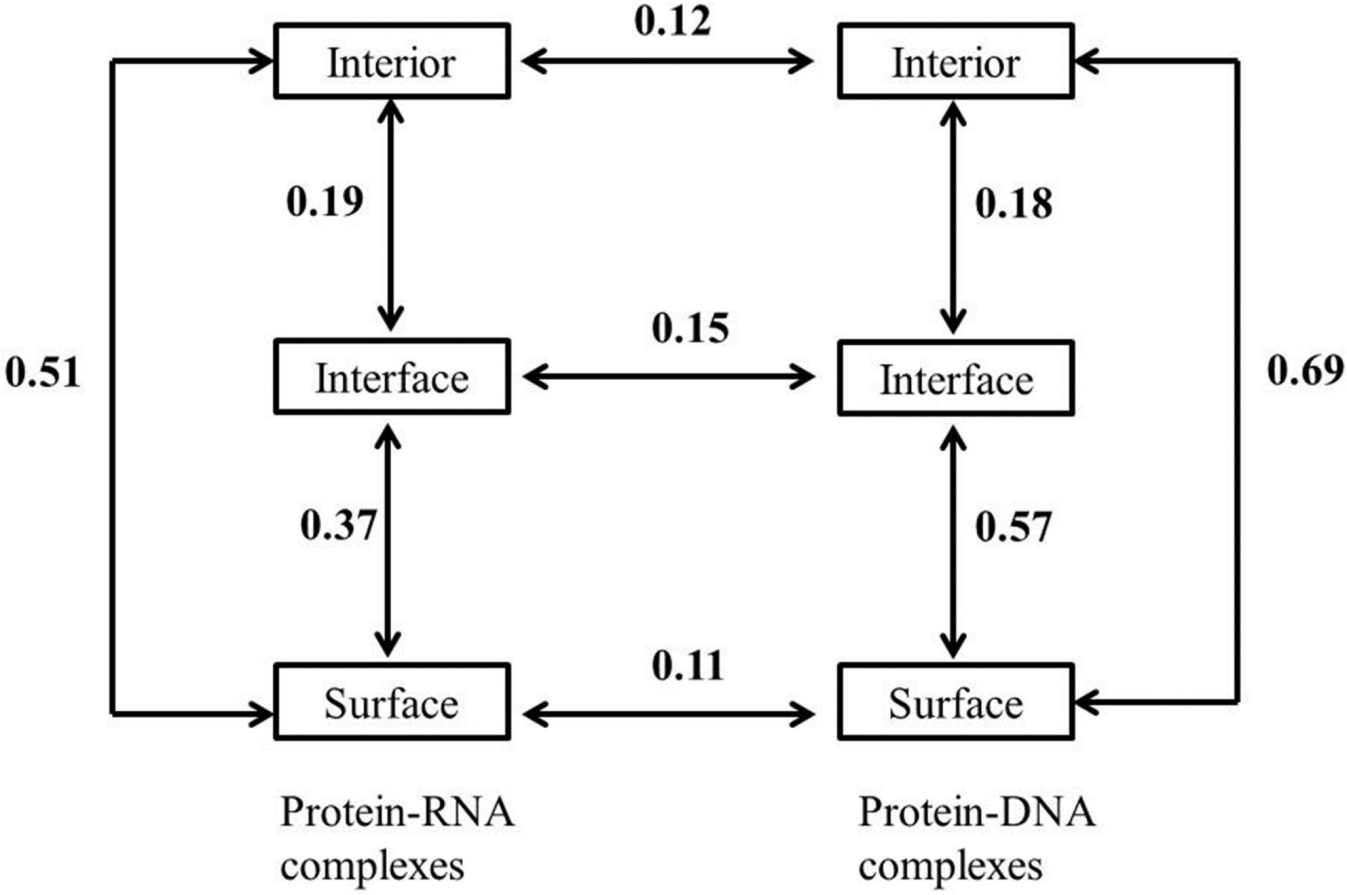
Euclidean distances (Δs) are calculated based on the mean normalized entropy <s> values of each amino acid residues.

The evolutionary distance between the different types of surfaces is calculated using the following Euclidean distance Δs:

(3) \begin{equation*} \Delta s^2 = \frac{1}{{19}}\sum\nolimits_{i = 1,20} {\left( { < s >_i - < s >^\prime_i } \right)^2 } \end{equation*}

where <s>*_i_* and <s>*_i_′* are the values of <s> contributed by each residue type into two surfaces (interior/interface, interface/surface or interior/surface). Figure 5 confirms that the protein interior and the interface are evolutionary closely related, however, both of them are distantly related to the solvent exposed surface.

### Conservation of residues at multi-subunit interfaces

In protein–RNA complexes involving more than one polypeptide chain, an interface residue in a given chain can be present either in PP interface, or in PR interface or in both (PP+PR). Our data set contains 39 complexes with multiple polypeptide chains (a total of 97 chains containing 22 284 residues). In these 39 cases, residues in a polypeptide chain are subject to evolutionary pressure to bind a RNA molecule as well as to bind another polypeptide. All these residues are divided into above three categories depending on their appearance, and <s> is calculated for each of these three categories. Of all these residues, 22% are at the protein interior, 49% are at the solvent exposed surface, 20% are at the PP interface, 7% are at the PR interface and 2% are at the PP+PR interface. We find the residues at the PP+PR interfaces (<s> = 0.84) are conserved to similar extent to those at the PR interfaces (<s> = 0.85), while the residues at the PP interfaces (<s> = 0.99) are the least conserved.

**Figure 6.**
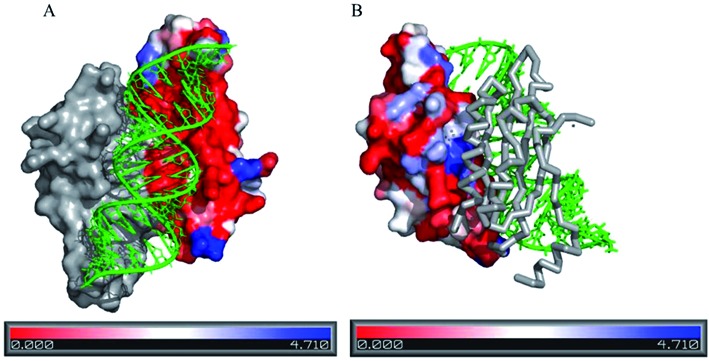
Conservation of amino acid residues in p19 complexed with siRNA (PDB id: 1R9F). Here, the p19 homodimer interacts with the duplex siRNA. (**A**) The interface between the protein and the RNA. (**B**) The homodimer interface between the two polypeptide chains of p19. The sequence entropies are mapped on the protein surface with the colour code provided at the bottom (red stands for the maximum conservation and blue stands for the minimum). The RNA backbone is shown in green cartoon. In panel B, one polypeptide chain is shown in surface, while the other one is shown in grey tube.

Figure 6 illustrates the degree of residue conservation at the protein surface involved in protein–RNA recognition along with the protein–protein interaction. Here, the two homodimeric chains of p19 recognize a duplex siRNA (PDB id: 1R9F). The protein surface recognizing the RNA is uniformly red, indicating highly conserved region (<s> = 0.60). In contrast, the protein surface in contact with the dimeric interface undergoes frequent mutations (<s> = 1.77). In this complex, the most conserved residues (<s> = 0.36) are the ones, which are present in both PP and PR interfaces.

### Relative conservation of residues interacting with the major and the minor grooves

Conservation of amino acid residues interacting with the major or the minor groove of the RNA have been analysed in a subset containing 43 complexes with duplex RNA. A residue is assigned to interact with the major groove if it makes at least one H-bond with one of the major groove atoms (N6/O6, N7 atoms of purines and O4, N4 atoms of pyrimidines). Similarly, a residue is assigned to interact with the minor groove if it makes at least one H-bond with one of the minor groove atoms (N2, N3 atoms of purines and O2 of pyrimidines). In all the above cases, the H-bond interactions between the amino acid residues and the Watson-Crick paired bases are considered. In our data set, we find 40 residues interacting with the major groove and 27 with the minor groove. Among the different residues interacting with the major groove, 55% are positively charged (Arg, Lys), 32% are neutral polar (Asn, Gln, His, Ser, Thr, Tyr, Trp, Cys, Met) and 13% are negatively charged (Asp, Glu). We find none of the hydrophobic residues interact with the major groove. Among the residues interacting with the minor groove, 34% are positively charged, 51% are neutral polar, 7% are negatively charged and 8% are hydrophobic (Gly, Ala, Pro, Val, Ile, Leu, Phe). We find the residues recognizing the major groove have an average <s> of 0.71, while those recognizing the minor groove have an average <s> of 0.89. These findings indicate that the residues involved in the recognition of the major groove of a duplex RNA are relatively better conserved than those involved in the recognition of the minor groove.

### Conservation of residues at preserved, hydrated and dehydrated sites

Water molecules play important roles in protein–RNA recognition (29). Comparing the bound and the unbound structures of the protein–RNA complexes, we identified three possible hydration sites at the interface: (i) water preservation site (WP), where a protein polar group makes the same H-bond with a water molecule in the bound and in the unbound structures; (ii) hydrated site (WH), where a protein polar group in the bound structure makes H-bond(s) with a water molecule, which is absent in the unbound structure; and (iii) dehydrated site (WD), where a protein polar group in the unbound structure makes H-bond(s) with a water molecule, which is absent in the bound structure. Following Barik and Bahadur (29), we found 38 complexes having unbound structures of the protein component. All these complexes contain water molecules in their bound and the unbound structures. However, we discarded three of them as they do not satisfy the criteria set in the ‘Materials and Methods’ section. In 35 complexes, we find 271 residues at the WP site, 217 at the WH site and 4627 at the WD site. The distribution of different types of residues in three different sites is given in Table 2. It shows that the neutral polar residues behave similarly in all the three different sites. Hydrophobic residues are frequently found at the WD site compared to the WH and the WP sites. On the other hand, positively charged residues are frequently found at the WH site compared to the WP and the WD sites, while negatively charged residues are frequently found at the WP site compared to the WD and the WH sites. Sequence entropy was used to quantify the relative conservation of the residues at the three different sites. We find that the residues present at the WP site are the most conserved (<s> = 0.66) compared to those present at the WH (<s> = 0.87) and at the WD (<s> = 1.01) sites (*P* = 2.8E-13).

**Table 2.** Percentage contribution of residues in three sites

Classification^a^	WP site	WH site	WD site
Positively charged	23	25	15
Negatively charged	21	12	17
Neutral polar	36	36	36
Hydrophobic	20	27	32

^a^Positively charged: Arg, Lys. Negatively charged: Asp, Glu. Neutral polar: Asn, Gln, Ser, Thr, His, Trp, Tyr, Cys, Met. Hydrophobic: Phe, Ile, Leu, Val, Pro, Ala, Gly.

### Prediction of the binding hot spots

Alanine scanning mutagenesis data were curated from the literature for 80 single mutations in 13 protein–RNA complexes. They are presented in Supplementary Table S3. Majority of the mutated residues (65/80) are involved in RNA recognition, while 14 are found at the protein surface and one at the protein interior. The ΔΔG varies between −1.35 kcal/mol and 4.03 kcal/mol (Table 3). Out of 65 mutations involving RNA recognition, 57 are destabilized and only eight are stabilized mutations. Of these destabilizing mutations, fewer than half (42%) cause significant loss in binding affinity (ΔΔG ≥ 1.0). The greatest reduction in binding affinity (ΔΔG ≥ 2.0 kcal/mol) were found for the substitution of three residues: F127A (2.09 kcal/mol) in aspartyl-tRNA synthetase complexed with tRNA(Asp) (PDB id: 1ASY); R510A (3.59 kcal/mol) and W508A (4.03 kcal/mol) in an elongation factor bound to mRNA complex (PDB id: 2PJP). All these three residues are evolutionary conserved as reflected by their normalized entropy (<s>) of 0.26, 0.45 and 0.47, respectively. Large effects in the binding affinity (1.0 < ΔΔG ≤ 2.0) were also observed for 20 mutations with <s> = 0.60; however, the correlation between binding affinity and sequence entropy is poor (*R* = 0.05).

**Table 3.** Change in binding free energy (ΔΔG) obtained through alanine mutagenesis

Class	I	II	III	IV	V
ΔΔG (kcal/mol)	≤−1.0	<−1.0 to ≤0.2	<0.2 to ≤1.0	<1.0 to ≤2.0	>0.2
Range	−1.35	−0.52 to 0.16	0.21 to 1.00	1.06 to 1.81	2.09 to 4.03
Average	-	−0.08	0.60	1.39	3.24
Number of mutants					
total	1	12	29	20	3
training set	1	8	22	13	2
test set	-	4	7	7	1
Entropy (<s>)					
Range	0.31	0 to 1.49	0 to 3.13	0 to 1.79	0.26 to 0.47
Average	0.31	0.49	0.77	0.61	0.39

^a^ΔΔG was calculated according to the formula: $\Delta \Delta {\rm G} = {\rm RT}\ln \frac{{{\rm K}_{\rm d} {\rm mutant}}}{{{\rm K}_{\rm d} {\rm WT}}}$.

**Figure 7.**
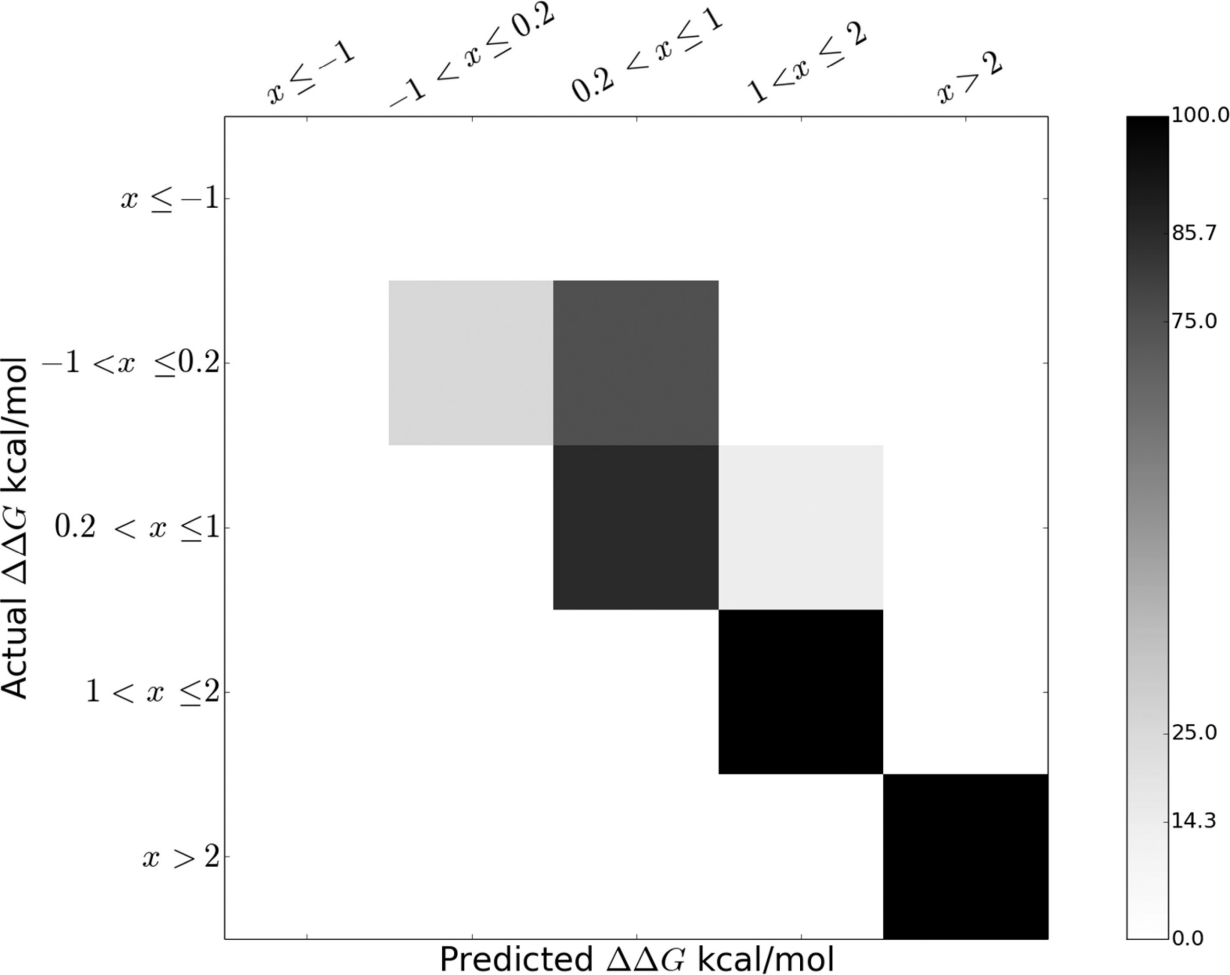
Confusion matrix representing the quality of ΔΔG predictions using RF. Diagonal values represents the correct predictions. Values outside the diagonal are misclassified. The scale represents the percentage of instances.

We used RF to predict the class of ΔΔG by using the structural and physicochemical attributes of the interface. All these parameters for each mutation are given in the Supplementary Table S4. We obtained prediction accuracy of 80% as shown in the confusion matrix (Figure 7). We have only one instance in class I, which is considered in the training set. Out of four instances in class II, only one is predicted correctly. Of the three mispredictions, the mutated residue is missing in the unbound structure in one instance (PDB id: 1YVP, K136A) resulting in missing values of structural parameters (Supplementary Table S4), in the second instance (PDB id: 2ZZM, R181A), the misprediction may be due to the large conformational change of Arg (C_α_ rmsd 8.8 Å) between the bound and the unbound structures, and in the last case (PDB id: 2Y8W, R27A), the mutated Arg is fully conserved (<s> = 0), however, the mutation resulted into a stabilized one (ΔΔG = −0.16). Of the 7 instances in class III, only one is mispredicted, while all the instances in other two classes are predicted accurately. Although the model generated mispredicted instances, they are all close to their actual class.

We have implemented this method in a web server ‘HotSPRing’ (http://www.csb.iitkgp.ernet.in/applications/HotSPRing/main), which is freely accessible. Users need to submit a protein–RNA complex file in PDB format, and their respective chain identifiers along with the structural homologs derived from the HSSP alignment. The unbound conformation of the protein is also expected but is not mandatory. Out of nine parameters we have used to train the prediction model, two parameters, ΔASA and C_α_-rmsd, are generated by using the unbound structure. The necessary documentation is provided in the web application. Upon successful completion of processing, the user is provided with a file containing the details of predicted range of ΔΔG for each of the RNA binding residues (RBRs) in the submitted protein–RNA complex.

## DISCUSSION

Sequence entropy, quantified in terms of Shannon's formula (21), is well established to identify the functionally important residues at the macromolecular binding sites. It has been widely used to study the protein–protein interfaces, as well as to identify the protein–protein binding sites (2,7–10,12). This study investigates the evolution of residues at the protein–RNA recognition sites, and compares with those residues present at the protein interior and at the protein surface. Moreover, we identified special kind of residues, which are part of the protein–protein as well as the protein–RNA interfaces in complexes where multiple polypeptide chains are involved in RNA recognition. These residues are termed as the multi-interface residues, and their evolution is also studied. Comparing the bound and the unbound structures of the components involved in protein–RNA complexes, we identified three sites at the protein surface that interact with the interface waters: water preservation site (WP), hydrated site (WH) and dehydrated site (WD). The analysis has been extended to the protein–DNA complexes taken from Setny et al., (30), and a comparative study has been performed to understand the evolution of the two types of interfaces. Finally, by combining sequence entropy with other structural and physicochemical attributes of the interfaces, a model is developed for efficient prediction of the binding hot spots at the protein–RNA recognition sites.

Analysis of 203 polypeptide chains taken from 145 protein–RNA complexes shows that some of the RBPs evolve faster compared to others. This observation is true among the complexes within the different classes as well as across the classes. Moreover, for RBPs having multiple polypeptide chains, each chain can evolve independently. This is exemplified in the complex between 16S rRNA and ribosomal proteins S6, S15 and S18 (PDB id: 1G1X). Although the three proteins interact with the same 16S rRNA, their degree of conservation varies. S15 evolves very slowly with <S> of 0.08, while S18 and S6 evolve relatively faster with <S> of 0.46 and 0.83, respectively.

The mean normalized sequence entropy shows that the residues at the protein–RNA interfaces are better conserved than those at the surfaces exposed to the solvent molecules. Moreover, interface residues experience almost similar evolutionary pressure as those present at the protein interior. Similar findings are observed in the protein–DNA interfaces (Table 1). The findings of the present study is in agreement with the previous analysis performed by Spriggs and Jones (31), showing that the RBRs are better conserved than other surface residues. Furthermore, our findings are in line with the studies on protein–protein complexes, which show that the interfaces are better conserved than the rest of the protein surface, and the evolutionary pressure on the residues at the protein interior is almost similar to those on the protein–protein interfaces (7–10,12,32).

Many protein–RNA complexes are stabilized by additional protein–protein interactions involving an auxiliary protein, or via dimerization of the RBDs (15). In these complexes, residues in a given polypeptide chain can be simultaneously present in the PP and in the PR interfaces. We identify these residues as highly conserved compared to the residues which are present either in the PP interfaces or in the PR interfaces. Multi-interface residues play an important role in the overall stability of the protein–RNA complexes (15), and they may be probed for their contribution to the stability of the complexes. Bahadur and Janin (11) also observed the important roles played by the multi-interface residues in viral capsid assemblies. They found that the degree of conservation of an amino acid residue increases with its presence in multiple interfaces, and the constraint imposed by each adjacent subunit interface on the polypeptide sequence are additive to some extent.

We show that the residues involved in H-bond interactions with the Watson-Crick paired bases at the RNA major groove are better conserved than those involved in H-bond interactions with the minor groove. The RNA minor groove is wide and shallow, hence more accessible to the amino acid residues. On the other hand, the major groove is narrow and deep, thus less accessible to the amino acid residues. This allows minor groove to make optimal van der Waals interactions, atomic packing, extensive H-bonding and hydrophobic surface burial, thereby creating energetically favourable interactions with the amino acid residues (33). This provides a favourable environment to the residues interacting with the minor groove, and they are subject to less evolutionary pressure.

We find that the residues at the WP site are better conserved than the residues at the WH or at the WD sites. At the WP sites, the same water-protein H-bond is found at the unbound and the corresponding bound structure. Hence, the residues at this site tend to be conserved during evolution. Compared to the WP site, the WH and the WD sites are relatively random. Hence, the residues at these sites experience less evolutionary constrain, allowing them to mutate frequently. Water molecules play an important role in the stability of macromolecular recognition (29,34), and including them may improve the models for the prediction of the binding hot spots (6).

In spite of the diversity of protein–RNA recognition sites, the simple RF model used in this study shows significant prediction efficiency of the change in binding free energy obtained through experimental alanine-scanning mutagenesis. Remarkably, our RF model predicts 80% of the instances of ΔΔG correctly using the sequence and structural parameters involving sequence entropy, H-bonds, salt-bridges, stacking interactions, atomic packing, change in solvent accessibility and backbone conformation upon binding, and change in the chemical properties of residues upon mutation. All the mispredictions are close to their actual class. Moreover, all the instances with ΔΔG > 1.0 kcal/mol (plausible hot-spots) are predicted accurately in their respective classes. Deriving binding affinity from the structural attributes of the macromolecules is a difficult task as they do not follow a simple correlation, and the prediction becomes almost impossible when large conformation change is associated with the complex formation (35). We find significant correlation between ΔΔG and C_α_ rmsd (*R* = 0.5) only in class II. Besides, we find significant contribution of the atomic packing (represented in terms of LD) in binding affinity. LD is positively correlated (*R* = 0.6) with ΔΔG in class II, majority of which are stabilized mutations. On the other hand, LD is inversely correlated with ΔΔG in class III and IV (*R* = −0.3 and −0.5, respectively), which are generally destabilized mutations. This signifies that atomic packing plays an important role in binding affinity. We also observe significant change in binding affinity when the evolutionary conserved residues are mutated (class V). These results suggest that the information derived from structure and sequence can be efficiently used to predict the binding hot spots at the protein–RNA recognition sites. Kortemme and Baker (6) also showed that the structural information is useful in prediction of binding hot spots at protein–protein interfaces. Although our model fails to predict in 20% instances, yet they are predicted close to their actual class.

## CONCLUSION

This study shows the relative conservation of amino acid residues involved in RNA recognition in protein–RNA complexes. Besides, we identified multi-interface residues that participate simultaneously in protein–protein and protein–RNA interfaces in complexes involving multiple polypeptide chains. These multi-interface residues are highly conserved compared to any other residues. It would be interesting to study the effect of mutation of these residues on the stability of the complex. Owing to the functional diversity of protein–RNA complexes, their interfaces have been increasingly used as the target site for the inhibitor development. Although the number of available mutations is small, we believe that the method developed for the prediction of binding hot spots at the protein–RNA recognition site will set a platform for further development when large number of mutation data will be available in this field. Additionally, the method for hot spot prediction described in this study could be effectively used to narrow down the target residues for inhibiting protein–RNA interactions. Our method could also be useful for engineering the protein–RNA interfaces with desired binding affinity.

## Supplementary Material

SUPPLEMENTARY DATA
