# Supplementary material for: Probing binding hot spots at protein–RNA recognition sites
Source: Nucleic Acids Res. 2015 Sep 13;44(2):e9. doi: 10.1093/nar/gkv876 (PMC4737170; doi:10.1093/nar/gkv876)
Supplement: SUPPLEMENTARY DATA [file supp_44_2_e9__index.html]

Probing binding hot spots at protein–RNA recognition sites — Probing binding hot spots at protein–RNA recognition sites — SUPPLEMENTARY DATA 

# Probing binding hot spots at protein–RNA recognition sites

## SUPPLEMENTARY DATA

- SUPPLEMENTARY DATA
