# Supplementary material for: Probing binding hot spots at protein–RNA recognition sites
Source: Nucleic Acids Res. 2015 Sep 13;44(2):e9. doi: 10.1093/nar/gkv876 (PMC4737170; doi:10.1093/nar/gkv876)
Supplement: SUPPLEMENTARY DATA [file supp_gkv876_nar-02005-met-n-2015-File002.docx]

Supplementary Table S1: Dataset of 145 protein-RNA complexes (classified into four different class based on the type of RNA associated with the protein).

| PDB Id^a^ | Composition^b^ | Res^c^ | Pro Chid^d^ | Chain Length^e^ | Aligned  Sequences^f^ | <S>^g^ |
| --- | --- | --- | --- | --- | --- | --- |
| 1. **tRNA (30)** | | | | | | |
| 1ASY | Yeast aspartyl-tRNA synthetase | 2.9 | A | 490 | 225 | 1.25 |
| 1B23 | *T. Aquaticus* EF-TU | 2.6 | P | 405 | 2270 | 0.63 |
| 1C0A | *E. coli* aspartyl-tRNA synthetase | 2.4 | A | 587 | 1500 | 1.06 |
| 1F7U | Arginyl-tRNA synthetase | 2.2 | A | 608 | 84 | 1.06 |
| 1FFY | Isoleucyl-tRNA synthetase | 2.2 | A | 917 | 564 | 1.16 |
| 1GAX | Valyl-tRNA synthetase | 2.9 | A | 866 | 854 | 1.25 |
| 1H3E | *T. thermophilus* tyrosyl-tRNA synthetase | 2.9 | A | 429 | 506 | 1.07 |
|  |  |  | A' | 429 | 506 | 1.07 |
| 1H4S | *T. thermophilus* prolyl-tRNA synthetase | 2.9 | A | 475 | 478 | 1.28 |
|  |  |  | B | 475 | 478 | 1.28 |
| 1J1U | *M. jannaschii* Tyrosyl-tRNA synthetase | 2.0 | A | 302 | 116 | 1.09 |
|  |  |  | A' | 302 | 116 | 1.09 |
| 1N78 | *T. thermophilus* glutamyl-tRNA synthetase | 2.1 | A | 468 | 144 | 1.17 |
| 1QF6 | *E. coli* threonyl-tRNA synthetase | 2.9 | A | 641 | 1019 | 1.06 |
| 1QTQ | *E. Coli* Glutaminyl-tRNA synthetase | 2.3 | A | 532 | 1778 | 1.18 |
| 1SER | *T. thermophilus*seryl-tRNA synthetase | 2.9 | A | 374 | 58 | 1.15 |
|  |  |  | B | 421 | 58 | 0.65 |
| 1U0B | *E.coli* cysteinyl-tRNA synthetase | 2.3 | B | 461 | 1304 | 1.2 |
| 1VFG | CCA-adding enzyme | 2.8 | B | 343 | 51 | 0.54 |
| 2AZX | *H.sapiens* tryptophanyl-tRNA synthetase | 2.8 | A | 393 | 306 | 1.11 |
|  |  |  | A' | 393 | 306 | 1.11 |
| 2CSX | Methionyl-tRNA synthetase | 2.7 | A | 465 | 1577 | 1.11 |
| 2DLC | Tyrosyl-tRNA synthetase | 2.4 | X | 339 | 182 | 1.02 |
|  |  |  | X' | 339 | 182 | 1.02 |
| 2DU3 | O-phosphoseryl-tRNA synthetase | 2.6 | A | 534 | 58 | 1.14 |
|  |  |  | A' | 534 | 58 | 1.14 |
|  |  |  | B | 534 | 58 | 1.14 |
|  |  |  | B' | 534 | 58 | 1.14 |
| 2FK6 | RNase Z | 2.9 | A | 307 | 193 | 1 |
| 2FMT | methionyl-tRNAfmet transformylase | 2.8 | A | 315 | 480 | 1.2 |
| 2ZM5 | isopentenyltransferase | 2.6 | A | 306 | 2174 | 0.78 |
| 2ZUE | Arginyl-tRNA synthetase | 2.0 | A | 628 | 18 | 0.76 |
| 2ZZM | Trm5 | 2.7 | A | 333 | 16 | 0.53 |
| 3ADB | O-phosphoseryl-tRNA kinase | 2.8 | A | 250 | 34 | 1.02 |
| 3AMT | Agmatinylcytidine synthetase TiaS | 2.9 | A | 417 | 15 | 0.9 |
| 3EPH | DMATase | 3.0 | A | 402 | 45 | 0.94 |
| 3HL2 | Selenium transferase | 2.8 | A | 445 | 115 | 0.86 |
|  |  |  | A' | 445 | 115 | 0.86 |
|  |  |  | B | 445 | 115 | 0.86 |
|  |  |  | B' | 445 | 115 | 0.86 |
| 3VJR | Peptidyl-tRNA hydrolase | 2.4 | A | 193 | 1139 | 0.8 |
| 3ZJT | E.coli leucyl-tRNA synthetase | 2.2 | A | 820 | 1929 | 0.81 |
| 1. **Ribosomal (10)** | | | | | | |
| 1DFU | Ribosomal Protein L25 | 1.8 | P | 94 | 455 | 0.96 |
| 1FEU | Ribosomal protein TL5 | 2.3 | A | 186 | 12 | 0.83 |
| 1G1X | ribosomal proteins S15, S6, S18 | 2.6 | A | 105 | 1545 | 0.08 |
|  |  |  | B | 88 | 1545 | 0.83 |
|  |  |  | C | 46 | 1545 | 0.46 |
| 1I6U | *M. jannaschii* ribosomal protein S8 | 2.6 | A | 130 | 442 | 1.11 |
| 1MJI | *T. thermophilus* ribosomal protein L5 | 2.5 | A | 181 | 3000 | 1.09 |
| 1MMS | *T. maritima* ribosomal protein L11 | 2.6 | A | 134 | 2999 | 1.05 |
| 1MZP | *S. acidocaldarius* ribosomal protein L1P | 2.7 | A | 217 | 13 | 0.45 |
| 1S03 | *E. coli* ribosomal protein S8 | 2.7 | H | 128 | 2975 | 1.17 |
| 1SDS | 50S ribosomal protein L7Ae | 1.8 | C | 112 | 320 | 0.94 |
| 2HW8 | Ribosomal protein L1 | 2.1 | A | 228 | 836 | 1.02 |
| 1. **Duplex (43)** | | | | | | |
| 1DI2 | dsRBD A | 1.9 | A | 71 | 125 | 0.93 |
|  |  |  | B | 60 | 125 | 0.85 |
| 1HQ1 | *E.coli* SRP | 1.5 | A | 77 | 1660 | 1.13 |
| 1MSW | T7 RNA polymerase | 2.1 | D | 865 | 102 | 0.71 |
| 1N35 | Reovirus polymerase | 2.5 | A | 1264 | 76 | 0.44 |
| 1OOA | NF-kappaB p50 | 2.5 | A | 313 | 173 | 1.25 |
| 1Q2R | Guanine transglycosylase | 2.9 | A | 376 | 2537 | 0.86 |
|  |  |  | B | 376 | 2537 | 0.86 |
| 1R3E | Pseudouridine Synthase TruB | 2.1 | A | 306 | 21 | 1.12 |
| 1R9F | p19 | 1.9 | A | 121 | 62 | 0.26 |
|  |  |  | A' | 121 | 62 | 0.26 |
| 1SI3 | PAZ domain of eIF2c1 | 2.6 | A | 121 | 354 | 0.94 |
| 1WNE | Foot-and-mouth disease virus polymerase | 3.0 | A | 476 | 278 | 0.42 |
| 1YVP | Ro autoantigen | 2.2 | B | 532 | 92 | 0.64 |
| 1ZBI | RNase H | 1.9 | A | 135 | 60 | 1.04 |
| 2AZ0 | Flock house virus B2 | 2.6 | A | 70 | 6 | 0.75 |
|  |  |  | B | 71 | 6 | 0.74 |
| 2BGG | A. fulgidus PIWI | 2.2 | A | 395 | 8 | 0.37 |
| 2GXB | Z alpha domain of adenosine deaminase | 2.3 | A | 62 | 90 | 0.94 |
| 2OZB | Ribonucleoprotein | 2.6 | A | 126 | 510 | 1 |
|  |  |  | B | 240 | 510 | 0.92 |
| 2PJP | *E.coli* SelB | 2.3 | A | 121 | 1281 | 0.48 |
| 2R8S | synthetic FAB | 2.0 | L | 214 | 167 | 0.98 |
|  |  |  | H | 218 | 167 | 1.25 |
| 2XD0 | *P. atrosepticum* ToxN | 3.0 | A | 162 | 7 | 0.14 |
| 2Y8W | Endoribonuclease Cse3 | 1.8 | A | 215 | 5 | 0.34 |
| 2YKG | RIG-I | 2.5 | A | 636 | 50 | 0.58 |
| 2ZI0 | TAV2b | 2.8 | A | 60 | 36 | 0.48 |
|  |  |  | B | 55 | 36 | 0.74 |
| 2ZKO | NS1 protein of influenza A | 1.7 | A | 70 | 2598 | 0.41 |
|  |  |  | B | 70 | 2598 | 0.41 |
| 3A6P | Exp-5:RanGTP | 2.9 | A | 1072 | 623 | 0.3 |
|  |  |  | C | 170 | 623 | 0.68 |
| 3BSN | Norwalk virus polymerase | 1.8 | A | 479 | 623 | 0.29 |
| 3BT7 | Methyltransferase TrmA | 2.4 | A | 369 | 2016 | 0.75 |
| 3DD2 | Thrombin | 1.9 | H | 260 | 131 | 1.12 |
| 3EQT | Helicase DHX58 | 2.0 | A | 142 | 39 | 0.59 |
|  |  |  | B | 142 | 39 | 0.59 |
| 3FTE | Methyltransferase KsgA | 3.0 | A | 233 | 8 | 1.01 |
| 3IAB | RNases P/MRP | 2.7 | A | 148 | 42 | 0.74 |
|  |  |  | B | 107 | 42 | 0.91 |
| 3KS8 | Polymerase cofactor VP35 | 2.4 | A | 124 | 23 | 0.17 |
|  |  |  | B | 124 | 23 | 0.17 |
| 3MOJ | Helicase dbpA | 2.9 | B | 75 | 405 | 0.8 |
| 3O3I | Hiwi1 PAZ domain | 2.8 | X | 108 | 166 | 0.93 |
| 3OIJ | Methyltransferase | 3.0 | A | 218 | 368 | 1 |
|  |  |  | B | 218 | 368 | 1 |
| 3OL6 | Poliovirus polymerase | 2.5 | A | 461 | 1664 | 0.64 |
| 3RW6 | Nuclear RNA export factor 1 | 2.3 | A | 245 | 174 | 0.88 |
| 3SNP | Iron regulatory protein 1 | 2.8 | A | 850 | 879 | 0.91 |
| 3ZC0 | *A. fulgidus* C3PO | 3.0 | A | 191 | 5 | 0.57 |
|  |  |  | A' | 191 | 5 | 0.57 |
|  |  |  | B | 191 | 5 | 0.57 |
|  |  |  | B' | 191 | 5 | 0.57 |
|  |  |  | C | 191 | 5 | 0.57 |
|  |  |  | C' | 191 | 5 | 0.57 |
|  |  |  | D | 191 | 5 | 0.57 |
|  |  |  | D' | 191 | 5 | 0.57 |
| 4ATO | *B. thuringiensis* ToxN | 2.2 | A | 168 | 19 | 0.09 |
| 4FVU | Exonuclease | 2.9 | A | 208 | 307 | 0.86 |
| 4IG8 | oligoadenylate synthetase 1 | 2.7 | A | 338 | 238 | 0.96 |
| 4ILL | endoribonuclease Cas6 | 2.5 | A | 278 | 16 | 0.48 |
|  |  |  | B | 278 | 16 | 0.48 |
| 4L8H | bacteriophage Qβ coat protein | 2.4 | A | 123 | 52 | 0.22 |
|  |  |  | B | 123 | 52 | 0.22 |
| 1. **Single-stranded (62)** | | | | | | |
| 1AV6 | Cap-specific mRNA methyltransferase | 2.8 | A | 292 | 119 | 0.91 |
| 1C9S | TRAP | 1.9 | L | 70 | 426 | 0.96 |
| 1CVJ | poly(A)-binding protein | 2.6 | A | 182 | 3740 | 0.35 |
| 1G2E | RRMdomain of the HuD protein | 2.3 | A | 167 | 263 | 0.83 |
| 1JBS | Restrictocin | 2.0 | A | 149 | 64 | 0.8 |
| 1JID | *H. sapiens* SRP19 | 1.8 | A | 114 | 72 | 0.99 |
| 1K8W | Pseudouridine synthase B | 1.9 | A | 303 | 450 | 0.99 |
| 1KNZ | Rotavirus NSP3 | 2.5 | A | 157 | 554 | 0.6 |
|  |  |  | B | 138 | 554 | 0.58 |
| 1KQ2 | Hfq | 2.7 | A | 62 | 635 | 0.85 |
|  |  |  | B | 61 | 635 | 0.83 |
|  |  |  | H | 62 | 635 | 0.83 |
|  |  |  | I | 62 | 635 | 0.85 |
|  |  |  | K | 61 | 635 | 0.83 |
|  |  |  | M | 62 | 635 | 0.83 |
| 1LNG | M. jannaschii SRP19 | 2.3 | A | 87 | 15 | 0.99 |
| 1M5O | U1 Snp | 2.2 | C | 93 | 440 | 1.12 |
| 1M8V | *P. abyssi* Sm PROTEIN | 2.6 | A | 73 | 141 | 1.17 |
|  |  |  | M | 73 | 141 | 1.17 |
| 1M8W | Pumilio-homology domain | 2.2 | A | 341 | 512 | 1.14 |
| 1UVI | phi6 RNA polymerase | 2.2 | A | 664 | 11 | 0.16 |
| 1WPU | HutP antitermination protein | 1.5 | A | 149 | 84 | 0.18 |
| 1WSU | Elongation Factor SelB | 2.3 | A | 130 | 149 | 0.12 |
| 1ZBH | exonuclease ERI1 | 3.0 | A | 290 | 34 | 0.86 |
|  |  |  | D | 290 | 34 | 0.86 |
| 1ZH5 | La autoantigen | 1.9 | A | 186 | 79 | 1.03 |
|  |  |  | B | 184 | 79 | 0.97 |
| 2A8V | RHO | 2.4 | B | 118 | 711 | 1.06 |
| 2ANR | Nova-1 KH1/KH2 domain | 1.9 | A | 157 | 209 | 0.74 |
| 2ASB | Nus A | 1.5 | A | 226 | 416 | 1.04 |
| 2B3J | TadA | 2.0 | A | 153 | 2783 | 1.01 |
|  |  |  | B | 153 | 2783 | 1.01 |
| 2BH2 | Methyltransferase RumA | 2.2 | A | 422 | 246 | 1.14 |
| 2BX2 | RNase E | 2.9 | L | 503 | 3431 | 0.67 |
| 2DB3 | DEAD-box helicase vasa | 2.2 | A | 420 | 250 | 1.07 |
| 2G4B | Splicing factor U2AF | 2.5 | A | 172 | 96 | 0.92 |
| 2GIC | VSV nucleocapsid | 2.9 | A | 425 | 170 | 0.59 |
| 2I82 | Pseudouridine synthase RluA | 2.1 | A | 220 | 1335 | 0.82 |
| 2IX1 | RNase II | 2.7 | A | 643 | 133 | 0.94 |
| 2J0S | Exon junction complex | 2.2 | A | 395 | 579 | 1.08 |
|  |  |  | C | 143 | 579 | 0.61 |
|  |  |  | D | 89 | 579 | 0.57 |
|  |  |  | T | 44 | 579 | 0.18 |
| 2JEA | *S. solfataricus* exosome | 2.3 | A | 274 | 198 | 0.59 |
|  |  |  | B | 234 | 198 | 1.22 |
| 2JLU | Serine protease subunit NS3 | 2.0 | A | 451 | 2396 | 0.37 |
| 2PY9 | Poly(rC)-binding protein 2 | 2.6 | B | 72 | 363 | 0.5 |
| 2Q66 | *S.cerevisiae* poly(A) polymerase | 1.8 | A | 519 | 131 | 1.09 |
| 2R7R | Rotavirus polymerase VP1 | 2.6 | A | 1073 | 512 | 0.18 |
| 2VNU | Exonuclease Rrp44 | 2.3 | D | 694 | 166 | 0.92 |
| 2XGJ | Mtr4 | 2.9 | A | 961 | 326 | 1.04 |
| 2XNR | Nab3-RRM | 1.6 | A | 75 | 76 | 0.91 |
| 2XS2 | Murine Dazl | 1.4 | A | 87 | 156 | 0.67 |
| 2XZO | Upf1 helicase | 2.4 | A | 613 | 314 | 0.8 |
| 3AEV | Dim2p | 2.8 | B | 177 | 150 | 1.19 |
| 3BX2 | PUF4 | 2.8 | A | 328 | 51 | 1.04 |
| 3D2S | MBNL1 ZnF3/4 | 1.7 | A | 68 | 398 | 0.46 |
| 3I5X | Mss116p | 1.9 | A | 509 | 9 | 0.79 |
| 3IEV | GTPase era | 1.9 | A | 302 | 19 | 0.57 |
| 3K5Q | FBF | 2.2 | A | 400 | 13 | 0.69 |
| 3MDG | CFI(m)25 | 2.2 | A | 210 | 199 | 0.76 |
|  |  |  | B | 210 | 199 | 0.76 |
| 3NMR | CUG-binding protein 1 | 1.9 | A | 175 | 249 | 0.51 |
| 3O8C | HCV NS3 helicase | 2.0 | A | 645 | 2475 | 0.18 |
| 3PF4 | CspB | 1.4 | B | 66 | 2590 | 0.88 |
| 3QJJ | RAMP Protein | 2.8 | A | 243 | 16 | 0.79 |
| 3R2C | NusB-NusE | 1.9 | A | 138 | 372 | 0.08 |
|  |  |  | J | 80 | 372 | 0.61 |
| 3RC8 | Helicase SUPV3L1 | 2.9 | A | 609 | 100 | 0.65 |
| 3T5N | Lassa virus nucleoprotein | 1.8 | A | 282 | 185 | 0.6 |
| 4E78 | HCV polymerase | 2.9 | A | 538 | 2477 | 0.33 |
| 4H5P | Nucleocapsid | 2.2 | A | 244 | 109 | 0.81 |
|  |  |  | B | 244 | 109 | 0.81 |
| 4HOR | IFIT | 1.9 | A | 482 | 147 | 1.01 |
| 4J1G | Nucleocapsid | 2.8 | A | 228 | 140 | 0.38 |
|  |  |  | B | 228 | 140 | 0.38 |
|  |  |  | C | 228 | 140 | 0.38 |
|  |  |  | D | 228 | 140 | 0.38 |
| 4J7M | Dom3Z | 1.5 | A | 358 | 54 | 0.56 |
| 4M59 | Chloroplast ppr10 | 2.5 | A | 687 | 72 | 0.99 |
|  |  |  | B | 683 | 72 | 0.99 |
| 4MDX | mRNA interferase MazF | 1.5 | A | 115 | 2017 | 0.8 |
|  |  |  | B | 115 | 2017 | 0.8 |
| 4N2Q | Thylakoid assembly 8 | 2.8 | A | 198 | 7 | 0.35 |

^a^PDB id of the protein-RNA complex

^b^Composition of the complex

^c^Resolution of the X-ray structure.

^d^Chain ids of the protein subunit in the PDB entry. Symmetry-related chains are primed (e.g. A' in 1J1U).

^e^Length of the protein chains

^f^Multiple sequence alignments of the protein chains obtained from the HSSP database (1)

^g^Mean entropy calculated for the entire polypeptide chain.

Supplementary Table S2: Mean normalized entropies <s> of amino acid residues

|  | protein-RNA | | | protein-DNA^a^ | | | Propensity^b^ | |
| --- | --- | --- | --- | --- | --- | --- | --- | --- |
| Amino acid residues | Interior | Interface | Surface | Interior | Interface | Surface | protein-RNA | protein-DNA |
| Ala | 0.8 | 0.9 | 1.28 | 0.70 | 0.85 | 1.38 | -0.35 | -0.48 |
| Arg | 0.4 | 0.71 | 1.08 | 0.25 | 0.60 | 1.08 | -0.42 | -0.59 |
| Asn | 0.65 | 0.83 | 1.28 | 0.56 | 0.59 | 1.36 | -0.43 | -0.84 |
| Asp | 0.46 | 0.74 | 1.08 | 0.33 | 0.59 | 1.22 | -0.38 | -0.73 |
| Cys | 0.83 | 0.63 | 1.07 | 0.71 | 0.50 | 1.05 | -0.53 | -0.74 |
| Gln | 0.57 | 0.86 | 1.28 | 0.41 | 0.64 | 1.45 | -0.40 | -0.82 |
| Glu | 0.54 | 0.78 | 1.19 | 0.4 | 0.61 | 1.28 | -0.42 | -0.74 |
| Gly | 0.53 | 0.55 | 0.84 | 0.44 | 0.43 | 0.87 | -0.42 | -0.70 |
| His | 0.6 | 0.78 | 1.12 | 0.37 | 0.65 | 1.31 | -0.36 | -0.70 |
| Ile | 0.9 | 0.84 | 1.23 | 0.82 | 0.86 | 1.29 | -0.38 | -0.41 |
| Leu | 0.74 | 0.81 | 1.06 | 0.64 | 0.73 | 1.04 | -0.27 | -0.35 |
| Lys | 0.38 | 0.83 | 1.22 | 0.38 | 0.70 | 1.30 | -0.39 | -0.62 |
| Met | 0.91 | 0.75 | 1.21 | 0.96 | 0.80 | 1.32 | -0.48 | -0.50 |
| Phe | 0.68 | 0.65 | 0.98 | 0.55 | 0.58 | 0.88 | -0.41 | -0.42 |
| Pro | 0.51 | 0.69 | 0.96 | 0.33 | 0.59 | 0.92 | -0.33 | -0.44 |
| Ser | 0.78 | 0.87 | 1.25 | 0.80 | 0.76 | 1.47 | -0.36 | -0.66 |
| Thr | 0.75 | 0.79 | 1.26 | 0.76 | 0.68 | 1.41 | -0.47 | -0.73 |
| Trp | 0.51 | 0.65 | 0.75 | 0.38 | 0.42 | 0.78 | -0.14 | -0.62 |
| Tyr | 0.62 | 0.73 | 0.97 | 0.53 | 0.53 | 0.99 | -0.28 | -0.62 |
| Val | 0.91 | 0.96 | 1.24 | 0.83 | 0.79 | 1.34 | -0.26 | -0.53 |

^a^Calculated on a dataset of 110 protein-DNA complexes taken from (2) satisfying the condition mentioned in Materials and methods section.

^b^*P_i_* = ln(*f_i_/f_i′_*) where *f_i_* is the mean normalized entropy for the i^th^ residue at the interface and *f_i′_* is the same at the protein surface.

Supplementary Table S3: K_d_ values for different mutants

| PDB Id^a^ | Mutant^b^ | Temp^d^ | K_d_^§^ | ∆G^e^ | ∆∆G^f^ | Reference |
| --- | --- | --- | --- | --- | --- | --- |
| 1ASY | WT | 310 | 3.00E-08 | -10.20 |  | (3) |
|  | D 210A | 310 | 3.28E-07 | -8.79 | 1.41 |  |
|  | E 188A | 310 | 4.24E-07 | -8.64 | 1.56 |  |
|  | E 202A | 310 | 1.65E-07 | -9.20 | 1.00 |  |
|  | E 327A | 310 | 2.00E-08 | -10.44 | -0.24 |  |
|  | F 127A | 310 | 1.05E-06 | -8.11 | 2.09 |  |
|  | H 334A | 310 | 2.70E-07 | -8.91 | 1.29 |  |
|  | K 142A | 310 | 1.29E-07 | -9.34 | 0.86 |  |
|  | K 155A | 310 | 1.10E-07 | -9.44 | 0.76 |  |
|  | K 180A | 310 | 4.00E-07 | -8.68 | 1.52 |  |
|  | K 553A | 310 | 2.80E-07 | -8.89 | 1.31 |  |
|  | N 117A | 310 | 1.35E-07 | -9.32 | 0.88 |  |
|  | N 227A | 310 | 1.20E-07 | -9.39 | 0.81 |  |
|  | N 328A | 310 | 8.50E-08 | -9.59 | 0.61 |  |
|  | Q 121A | 310 | 2.75E-07 | -8.90 | 1.30 |  |
|  | Q 138A | 310 | 3.37E-07 | -8.78 | 1.42 |  |
|  | R 119A | 310 | 2.69E-07 | -8.91 | 1.29 |  |
|  | S 181A | 310 | 1.80E-07 | -9.15 | 1.05 |  |
|  | S 301A | 310 | 5.00E-08 | -9.90 | 0.30 |  |
|  | S 329A | 310 | 1.20E-07 | -9.39 | 0.81 |  |
|  | S 423A | 310 | 2.50E-08 | -10.31 | -0.11 |  |
|  | T 124A | 310 | 1.25E-07 | -9.36 | 0.84 |  |
|  | T 230A | 310 | 3.30E-07 | -8.79 | 1.41 |  |
|  | T 331A | 310 | 2.00E-07 | -9.09 | 1.11 |  |
|  | T 424A | 310 | 1.20E-07 | -9.39 | 0.81 |  |
|  | S 280A | 310 | 3.00E-08 | -10.20 | 0.00 |  |
|  | Q 300A | 310 | 2.50E-08 | -10.31 | -0.11 |  |
|  | F 304A | 310 | 3.00E-08 | -10.20 | 0.00 |  |
|  | K 293A | 310 | 3.50E-07 | -8.76 | 1.45 |  |
|  | K 428A | 310 | 1.25E-07 | -9.36 | 0.84 |  |
| 1JBS | WT | 310 | 5.83E-09 | -11.17 |  | (4) |
|  | D 143A | 310 | 2.41E-09 | -11.69 | -0.52 |  |
|  | D 40 A | 310 | 8.33E-09 | -10.96 | 0.21 |  |
|  | H 49 A | 310 | 2.40E-08 | -10.33 | 0.84 |  |
|  | K 110A | 310 | 6.67E-08 | -9.73 | 1.44 |  |
|  | K 111A | 310 | 1.25E-07 | -9.36 | 1.81 |  |
|  | K 113A | 310 | 2.00E-08 | -10.44 | 0.73 |  |
|  | K 42 A | 310 | 9.25E-09 | -10.90 | 0.27 |  |
|  | Q 141A | 310 | 1.19E-08 | -10.75 | 0.42 |  |
|  | R 65 A | 310 | 1.38E-08 | -10.66 | 0.51 |  |
|  | T 52 A | 310 | 2.48E-08 | -10.32 | 0.85 |  |
|  | F 51 A | 310 | 1.00E-08 | -10.85 | 0.32 |  |
| 1U0B | WT | 298 | 2.70E-07 | -8.56 |  | (5) |
|  | N 351A | 298 | 6.22E-06 | -6.79 | 1.77 |  |
| 1YVP | WT | 277 | 5.20E-09 | -10.04 |  | (6) |
|  | K 136A | 277 | 4.80E-09 | -10.08 | -0.04 |  |
|  | R 184A | 277 | 3.08E-08 | -9.10 | 0.94 |  |
| 2BX2 | WT | 310 | 2.00E-07 | -9.09 |  | (7) |
|  | R 373A | 310 | 1.20E-07 | -9.39 | -0.30 |  |
|  | F 57 A | 310 | 4.30E-07 | -8.63 | 0.45 |  |
|  | F 67 A | 310 | 6.70E-07 | -8.37 | 0.71 |  |
| 2IX1 | WT | 277 | 6.48E-09 | -9.92 |  | (8) |
|  | E 390A | 277 | 8.70E-09 | -9.77 | 0.15 |  |
|  | E 542A | 277 | 5.00E-10 | -11.27 | -1.35 |  |
|  | F 358A | 277 | 9.90E-09 | -9.70 | 0.22 |  |
|  | R 500A | 277 | 1.09E-08 | -9.65 | 0.27 |  |
|  | Y 253A | 277 | 3.50E-08 | -9.04 | 0.88 |  |
|  | Y 313A | 277 | 1.71E-08 | -9.41 | 0.51 |  |
| 2PJP | WT | 298 | 3.00E-10 | -12.42 |  | (9) |
|  | R 510A | 298 | 1.70E-07 | -8.83 | 3.59 |  |
|  | V 509A | 298 | 3.60E-09 | -11.01 | 1.41 |  |
|  | W 508A | 298 | 3.70E-07 | -8.39 | 4.03 |  |
|  | D 511A | 298 | 1.10E-08 | -10.38 | 2.04 |  |
| 2XS2 | WT | 298 | 3.82E-08 | -9.67 |  | (10) |
|  | P 39 A | 298 | 1.53E-07 | -8.89 | 0.79 |  |
| 2Y8W | WT | 338 | 3.60E-09 | -12.49 |  | (11) |
|  | E 24 A | 338 | 2.30E-08 | -11.29 | 1.20 |  |
|  | E 38 A | 338 | 6.60E-09 | -12.10 | 0.39 |  |
|  | N 102A | 338 | 7.50E-09 | -12.01 | 0.48 |  |
|  | R 27 A | 338 | 2.80E-09 | -12.65 | -0.16 |  |
|  | S 34 A | 338 | 3.70E-09 | -12.47 | 0.02 |  |
| 2ZI0 | WT | 298 | 7.50E-08 | -9.29 |  | (12) |
|  | P 41 A | 298 | 7.60E-07 | -7.98 | 1.31 |  |
|  | W 50 A | 298 | 3.40E-07 | -8.43 | 0.86 |  |
| 2ZKO | WT | 298 | 1.25E-06 | -7.70 |  | (13) |
|  | R 37 A | 298 | 1.18E-06 | -7.73 | -0.03 |  |
|  | S 42 A | 298 | 1.20E-05 | -6.41 | 1.29 |  |
|  | T 49 A | 298 | 1.09E-05 | -6.47 | 1.23 |  |
|  | R 44 A | 298 | 4.94E-06 | -6.92 | 0.78 |  |
| 2ZZM | WT | 328 | 7.00E-07 | -8.83 |  | (14) |
|  | K 318A | 328 | 1.10E-06 | -8.55 | 0.28 |  |
|  | N 265A | 328 | 8.00E-07 | -8.75 | 0.08 |  |
|  | P 267A | 328 | 8.00E-07 | -8.75 | 0.08 |  |
|  | R 145A | 328 | 1.00E-06 | -8.61 | 0.22 |  |
|  | R 181A | 328 | 9.00E-07 | -8.68 | 0.15 |  |
|  | Y 177A | 328 | 1.10E-06 | -8.55 | 0.28 |  |
|  | R 186A | 328 | 1.20E-06 | -8.50 | 0.34 |  |
|  | D 223A | 328 | 1.00E-06 | -8.61 | 0.22 |  |
|  | K 137A | 328 | 1.60E-06 | -8.32 | 0.52 |  |
| 3EQT | WT | 298 | 1.05E-07 | -9.10 |  | (15) |
|  | E 573A | 298 | 2.13E-06 | -7.39 | 1.71 |  |
| 3MOJ | WT | 273 | 5.40E-11 | -12.26 |  | (16) |
|  | G 423A | 273 | 1.13E-10 | -11.88 | 0.38 |  |
|  | Y 407A | 273 | 1.28E-10 | -11.82 | 0.45 |  |

^a^PDB id of the protein-RNA complex.

^b^WT is for wildtype. The mutated residue name and residue number precedes the “Ala” one letter code.

^c^Temperature at which the binding constant was measured.

^d^The dissociation constant was taken from the literature.

^e^∆G was calculated according to the formula: ∆G = RTln(K_d_)

^f^∆∆G was calculated according to the formula: $\Delta\Delta\text{G}=\text{RTln}\frac{\text{K}_{\text{d}}\text{mutant}}{\text{K}_{\text{d}}\text{WT}}$

Supplementary Table S4: Different parameters used in prediction of ∆∆G

| PDBid^a^ | Mutant^b^ | Entropy^c^ | ∆∆G^d^ | HB^e^ | SB^f^ | C_α_-rmsd^g^ | ∆ASA^h^ | ∆ASA-SC^i^ | LD^j^ | Stacking^k^ | AA-var^l^ |
| --- | --- | --- | --- | --- | --- | --- | --- | --- | --- | --- | --- |
| 1ASY | E 327A | 0.16 | -0.24 | 1 | 0 | 1.37 | -27.37 | -21.55 | 43 | -1 | -1 |
| 1ASY | S 423A | 1.12 | -0.11 | 3 | 0 | 1.75 | -0.60 | 1.46 | 29 | -1 | -1 |
| 1ASY | S 301A | 0.19 | 0.30 | 0 | 0 | 1.93 | 7.48 | 7.93 | 25 | -1 | -1 |
| 1ASY | N 328A | 0.96 | 0.61 | 1 | 0 | 1.86 | -0.61 | -1.60 | 45 | -1 | -1 |
| 1ASY | K 155A | 0.86 | 0.77 | 3 | 2 | 0.73 | -5.88 | -5.90 | 16 | -1 | -1 |
| 1ASY | N 227A | 1.44 | 0.82 | 3 | 0 | 1.64 | -14.52 | -14.53 | 43 | -1 | -1 |
| 1ASY | S 329A | 0.16 | 0.82 | 2 | 0 | 2.27 | 17.65 | 15.43 | 68 | -1 | -1 |
| 1ASY | T 424A | 0.23 | 0.82 | 2 | 0 | 2.07 | 0.21 | -2.03 | 51 | -1 | -1 |
| 1ASY | T 124A | 1.02 | 0.84 | 3 | 0 | 1.42 | 1.43 | 1.43 | 36 | -1 | -1 |
| 1ASY | K 142A | 1.67 | 0.86 | 1 | 0 | 1.41 | 8.35 | 9.83 | 34 | -1 | -1 |
| 1ASY | N 117A | 1.56 | 0.89 | 2 | 0 | 0.68 | 27.29 | 26.85 | 47 | -1 | -1 |
| 1ASY | E 202A | 1.76 | 1.00 | 2 | 0 | 1.79 | 21.34 | 5.56 | 21 | -1 | -1 |
| 1ASY | S 181A | 0.84 | 1.06 | 3 | 0 | 1.30 | 9.37 | -3.95 | 42 | -1 | -1 |
| 1ASY | T 331A | 0.16 | 1.12 | 4 | 0 | 1.26 | 17.94 | 26.45 | 75 | -1 | -1 |
| 1ASY | R 119A | 0.36 | 1.29 | 2 | 1 | 1.58 | 26.21 | 27.74 | 58 | 1 | -1 |
| 1ASY | H 334A | 0.15 | 1.29 | 1 | 0 | 1.25 | 27.49 | 26.84 | 91 | 1 | -1 |
| 1ASY | Q 121A | 1.32 | 1.30 | 2 | 0 | 1.03 | -39.28 | -7.87 | 48 | -1 | -1 |
| 1ASY | K 553A | 1.00 | 1.32 | 1 | 2 | 0.95 | 24.65 | 11.72 | 37 | -1 | -1 |
| 1ASY | D 210A | 0.70 | 1.41 | 1 | 0 | 1.48 | 10.91 | 7.06 | 55 | -1 | -1 |
| 1ASY | T 230A | 0.67 | 1.41 | 2 | 0 | 1.21 | -12.79 | -14.56 | 45 | -1 | -1 |
| 1ASY | Q 138A | 0.34 | 1.42 | 2 | 0 | 1.48 | 7.03 | 7.09 | 54 | -1 | -1 |
| 1ASY | K 180A | 1.79 | 1.53 | 1 | 0 | 1.41 | -26.23 | -16.39 | 29 | -1 | -1 |
| 1ASY | E 188A | 0.22 | 1.56 | 5 | 0 | 1.75 | 7.36 | 7.36 | 42 | -1 | -1 |
| 1ASY | F 127A | 0.26 | 2.09 | 2 | 0 | 1.22 | 10.03 | 8.66 | 63 | 1 | 1 |
| 1JBS | D 143A | 0.97 | -0.52 | 2 | 0 | 0.95 | -4.77 | 3.46 | 25 | -1 | -1 |
| 1JBS | D 40 A | 0.96 | 0.21 | 2 | 0 | 0.24 | 2.89 | -1.37 | 32 | -1 | -1 |
| 1JBS | K 42 A | 0.96 | 0.27 | 1 | 1 | 0.79 | -67.89 | -52.19 | 29 | -1 | -1 |
| 1JBS | Q 141A | 0.94 | 0.42 | 0 | 0 | 2.06 | -24.54 | -18.73 | 20 | -1 | -1 |
| 1JBS | R 65 A | 0.96 | 0.51 | 1 | 3 | 0.77 | 0.15 | 4.40 | 20 | 1 | -1 |
| 1JBS | K 113A | 0.96 | 0.73 | 1 | 0 | 0.32 | -35.03 | -28.86 | 31 | -1 | -1 |
| 1JBS | H 49 A | 0.96 | 0.83 | 3 | 0 | 0.27 | 6.99 | 6.99 | 40 | 1 | -1 |
| 1JBS | T 52 A | 0.96 | 0.85 | 2 | 0 | 0.30 | -6.21 | -7.68 | 28 | -1 | -1 |
| 1JBS | K 110A | 0.88 | 1.44 | 3 | 0 | 1.08 | 16.59 | 6.60 | 27 | -1 | -1 |
| 1JBS | K 111A | 0.96 | 1.81 | 2 | 3 | 0.79 | -18.00 | -14.90 | 17 | -1 | -1 |
| 1U0B | N 351A | 0.34 | 1.78 | 4 | 0 | 0.38 | -5.36 | -5.36 | 30 | -1 | -1 |
| 1YVP | K 136A | 0.88 | -0.04 | 2 | 0 | - | - | - | 36 | -1 | -1 |
| 1YVP | R 184A | 0.78 | 0.94 | 4 | 1 | 0.89 | -7.19 | -6.57 | 56 | 1 | -1 |
| 2BX2 | R 373A | 0.76 | -0.30 | 2 | 2 | - | - | - | 35 | 1 | -1 |
| 2IX1 | E 542A | 0.31 | -1.35 | 2 | 0 | 0.81 | -1.09 | -0.51 | 35 | -1 | -1 |
| 2IX1 | E 390A | 0.20 | 0.16 | 3 | 0 | 1.02 | 9.08 | 9.53 | 57 | -1 | -1 |
| 2IX1 | F 358A | 0.20 | 0.22 | 0 | 0 | 1.56 | 9.87 | 6.89 | 62 | 2 | 1 |
| 2IX1 | R 500A | 0.20 | 0.27 | 5 | 3 | 0.50 | -6.47 | -6.45 | 65 | 1 | -1 |
| 2IX1 | Y 313A | 0.22 | 0.51 | 5 | 0 | 2.09 | 5.13 | 5.36 | 60 | 1 | -1 |
| 2IX1 | Y 253A | 0.22 | 0.89 | 1 | 0 | 0.50 | 17.16 | 16.05 | 42 | 2 | -1 |
| 2PJP | V 509A | 0.48 | 1.41 | 2 | 0 | - | - | - | 24 | -1 | 1 |
| 2PJP | R 510A | 0.45 | 3.59 | 4 | 0 | - | - | - | 24 | 2 | -1 |
| 2PJP | W 508A | 0.47 | 4.03 | 2 | 0 | - | - | - | 30 | 2 | 1 |
| 2Y8W | R 27 A | 0.00 | -0.16 | 6 | 1 | 0.44 | -16.38 | -15.39 | 61 | 1 | -1 |
| 2Y8W | S 34 A | 1.49 | 0.02 | 4 | 0 | 0.22 | 18.21 | 13.68 | 32 | -1 | -1 |
| 2Y8W | E 38 A | 3.13 | 0.39 | 3 | 0 | 0.31 | 1.60 | 1.91 | 23 | -1 | -1 |
| 2Y8W | N 102A | 0.00 | 0.47 | 2 | 0 | 0.41 | -0.73 | -0.73 | 75 | -1 | -1 |
| 2Y8W | E 24 A | 0.00 | 1.19 | 8 | 0 | 0.25 | -12.82 | -12.58 | 58 | -1 | -1 |
| 2ZI0 | W 50 A | 0.89 | 0.86 | 5 | 0 | - | - | - | 26 | 1 | 1 |
| 2ZI0 | P 41 A | 0.61 | 1.31 | 2 | 0 | - | - | - | 34 | -1 | 1 |
| 2ZKO | R 37 A | 0.35 | -0.03 | 4 | 0 | 0.72 | -35.38 | -35.76 | 29 | 1 | -1 |
| 2ZKO | T 49 A | 0.34 | 1.23 | 8 | 0 | 0.33 | -0.97 | -0.28 | 26 | -1 | -1 |
| 2ZKO | S 42 A | 0.99 | 1.28 | 10 | 0 | 0.38 | 5.90 | 7.03 | 47 | -1 | -1 |
| 2ZZM | N 265A | 0.00 | 0.08 | 1 | 0 | 9.23 | 0.59 | -2.02 | 42 | -1 | -1 |
| 2ZZM | P 267A | 0.00 | 0.08 | 0 | 0 | 11.75 | -2.45 | -3.04 | 55 | -1 | 1 |
| 2ZZM | R 181A | 0.00 | 0.16 | 4 | 5 | 8.76 | 8.17 | 13.91 | 78 | 1 | -1 |
| 2ZZM | R 145A | 0.00 | 0.22 | 4 | 0 | 8.30 | 50.27 | 27.75 | 51 | 1 | -1 |
| 2ZZM | K 318A | 0.00 | 0.28 | 2 | 1 | - | - | - | 81 | -1 | -1 |
| 2ZZM | Y 177A | 0.00 | 0.28 | 2 | 0 | 4.50 | 39.28 | 35.08 | 70 | 1 | -1 |
| 3EQT | E 573A | 0.00 | 1.70 | 6 | 0 | 1.14 | -14.82 | -11.34 | 32 | -1 | -1 |
| 3MOJ | G 423A | 0.21 | 0.38 | 1 | 0 | 0.41 | 10.97 | - | 56 | -1 | 1 |

^a^PDB id of the protein-RNA complex.
^b^The residue mutated with alanine.
^c^The normalised entropy of the mutated residue calculated from equation 2 described in the Materials and methods section.
^d^∆∆G values taken from Table S3. ^e^Number of hydrogen bonds were calculated using the HBPLUS program (17).
^f^Salt bridges were calculated following (18). To train the model the absolute values are converted into three groups based on the following: if a residue eligible to make salt-bridge but do not participate is assigned 1, a residue eligible and participate in salt-bridge is assigned 2; and a residue not eligible is assigned -1.
^g^C_α_-rmsd was calculated by superposing bound and unbound structures.
Change in SASA of a residue in the bound structure compared to its unbound form for the whole residue^h^ as well as for the side chain^i^. ^j^Local density index was calculated following (19).
^k^Stacking interactions were calculated following (20). Residues which are eligible for stacking interactions but not participate are assigned 1; residue eligible and participate in stacking interactions are assigned 2; and residues that are not eligible are assigned -1.
^l^Classification of mutation based on change in side chain property. If the mutation causes the change in hydrophobicity we assign a value -1 (e.g. E to A), otherwise 1.

Missing values (-) represents the unavailability of the unbound structures.

**References**

1. Schneider, R., de Daruvar, A. and Sander, C. (1997) The HSSP database of protein structure-sequence alignments. *Nucleic Acids Research*, **25**, 226-230.

2. Setny, P., Bahadur, R. and Zacharias, M. (2012) Protein-DNA docking with a coarse-grained force field. *BMC Bioinformatics*, **13**, 228.

3. Eriani, G. and Gangloff, J. (1999) Yeast aspartyl-tRNA synthetase residues interacting with tRNA(Asp) identity bases connectively contribute to tRNA(Asp) binding in the ground and transition-state complex and discriminate against non-cognate tRNAs. *J Mol Biol*, **291**, 761-773.

4. Plantinga, M.J., Korennykh, A.V., Piccirilli, J.A. and Correll, C.C. (2011) The Ribotoxin Restrictocin Recognizes Its RNA Substrate by Selective Engagement of Active Site Residues. *Biochemistry*, **50**, 3004-3013.

5. Hauenstein, S., Zhang, C.M., Hou, Y.M. and Perona, J.J. (2004) Shape-selective RNA recognition by cysteinyl-tRNA synthetase. *Nat Struct Mol Biol*, **11**, 1134-1141.

6. Stein, A.J., Fuchs, G., Fu, C., Wolin, S.L. and Reinisch, K.M. (2005) Structural insights into RNA quality control: the Ro autoantigen binds misfolded RNAs via its central cavity. *Cell*, **121**, 529-539.

7. Callaghan, A.J., Marcaida, M.J., Stead, J.A., McDowall, K.J., Scott, W.G. and Luisi, B.F. (2005) Structure of Escherichia coli RNase E catalytic domain and implications for RNA turnover. *Nature*, **437**, 1187-1191.

8. Barbas, A., Matos, R.G., Amblar, M., Lopez-Vinas, E., Gomez-Puertas, P. and Arraiano, C.M. (2008) New insights into the mechanism of RNA degradation by ribonuclease II: identification of the residue responsible for setting the RNase II end product. *J Biol Chem*, **283**, 13070-13076.

9. Soler, N., Fourmy, D. and Yoshizawa, S. (2007) Structural Insight into a Molecular Switch in Tandem Winged-helix Motifs from Elongation Factor SelB. *Journal of Molecular Biology*, **370**, 728-741.

10. Jenkins, H.T., Malkova, B. and Edwards, T.A. (2011) Kinked β-strands mediate high-affinity recognition of mRNA targets by the germ-cell regulator DAZL. *Proceedings of the National Academy of Sciences*, **108**, 18266-18271.

11. Sashital, D.G., Jinek, M. and Doudna, J.A. (2011) An RNA-induced conformational change required for CRISPR RNA cleavage by the endoribonuclease Cse3. *Nat Struct Mol Biol*, **18**, 680-687.

12. Chen, H.Y., Yang, J., Lin, C. and Yuan, Y.A. (2008) Structural basis for RNA‐silencing suppression by Tomato aspermy virus protein 2b. *EMBO reports*, **9**, 754-760.

13. Cheng, A., Wong, S.M. and Yuan, Y.A. (2009) Structural basis for dsRNA recognition by NS1 protein of influenza A virus. *Cell Res*, **19**, 187-195.

14. Christian, T., Lahoud, G., Liu, C., Hoffmann, K., Perona, J.J. and Hou, Y.M. (2010) Mechanism of N-methylation by the tRNA m1G37 methyltransferase Trm5. *RNA (New York, N.Y.)*, **16**, 2484-2492.

15. Li, X., Ranjith-Kumar, C.T., Brooks, M.T., Dharmaiah, S., Herr, A.B., Kao, C. and Li, P. (2009) The RIG-I-like Receptor LGP2 Recognizes the Termini of Double-stranded RNA. *Journal of Biological Chemistry*, **284**, 13881-13891.

16. Hardin, J.W., Hu, Y.X. and McKay, D.B. (2010) Structure of the RNA Binding Domain of a DEAD-Box Helicase Bound to Its Ribosomal RNA Target Reveals a Novel Mode of Recognition by an RNA Recognition Motif. *J Mol Biol*, **402**, 412-427.

17. McDonald, I.K. and Thornton, J.M. (1994) Satisfying hydrogen bonding potential in proteins. *Journal of molecular biology*, **238**, 777-793.

18. Xu, D., Tsai, C.J. and Nussinov, R. (1997) Hydrogen bonds and salt bridges across protein-protein interfaces. *Protein Engineering*, **10**, 999-1012.

19. Bahadur, R.P., Zacharias, M. and Janin, J. (2008) Dissecting protein–RNA recognition sites. *Nucleic acids research*, **36**, 2705-2716.

20. Allers, J. and Shamoo, Y. (2001) Structure-based analysis of protein-RNA interactions using the program ENTANGLE. *Journal of molecular biology*, **311**, 75-86.
